# Apical Ballooning of Takotsubo Cardiomyopathy in a Patient with Non-ST Elevation Myocardial Infarction Due to Right Coronary Artery Stenosis: The Trapped Octopus Got a Heart Attack

**DOI:** 10.7759/cureus.4415

**Published:** 2019-04-09

**Authors:** Mustafa Ajam, Adel Elmoghrabi, Said Ashraf, Ahmed Yassin, Mohamed Shokr

**Affiliations:** 1 Internal Medicine, Detroit Medical Center - Wayne State University, Detroit, USA; 2 Cardiology, Detroit Medical Center - Wayne State University, Detroit, USA

**Keywords:** takotsubo cardiomyopathy, acute coronary syndrome, acute coronary syndrome induced takotsubo, right coronary artery

## Abstract

Takotsubo cardiomyopathy (TCM) is characterized by apical ballooning with basal preservation in the absence of obstructive coronary artery disease (CAD) that can otherwise explain wall motion abnormalities. However, there is increasing evidence that acute coronary syndromes (ACSs) may coexist with TCM. This report describes a 61-year-old man with a previous medical history of hypertension, diabetes mellitus, and hyperlipidemia, who presented with acute chest pain and associated shortness of breath. He was diagnosed with a non-ST segment myocardial infarction. Echocardiography revealed impaired systolic function with evidence of apical and periapical ballooning of the left ventricle, characteristic of TCM. Coronary angiography revealed evidence of significant luminal stenosis of the right coronary artery (RCA), necessitating intervention with a drug-eluting stent. This patient demonstrated wall motion abnormalities characteristic of TCM beyond the territory of the affected coronary artery suggesting that CAD and TCM can coexist.

## Introduction

Apical ballooning with basal preservation in the absence of coronary artery obstruction is the hallmark of the most common variant of Takotsubo cardiomyopathy (TCM). Although the clinical presentation of TCM is similar to that of acute coronary syndrome (ACS), TCM is associated with a better prognosis, with reversal of left ventricular dysfunction occurring within weeks to months [1]. Current diagnostic criteria have emphasized the absence of significant coronary artery disease (CAD) that can otherwise explain the wall motion abnormalities seen in TCM. Increasing evidence has suggested, however, that TCM and ACS can coexist, especially in patients with new resting wall motion abnormalities beyond the territory of the affected coronary artery [2-3]. This report describes a patient with ACS likely due to right coronary artery (RCA) stenosis who was found to have coexistent TCM.

## Case presentation

A 61-year-old man with a previous medical history of hypertension, diabetes mellitus, and hyperlipidemia presented with acute pressure-like chest pain and shortness of breath. Physical examination revealed a heart rate of 71 beats per minute (bpm), a blood pressure of 144/71 mmHg, and an oxygen saturation of 96%. On cardiopulmonary examination, he had a normal S1 and S2 with no appreciable murmur, rub, or gallop and with clear lung fields bilaterally. Peripheral pulsations were 2+ and symmetric, and there was no evidence of lower extremity edema. His family history was notable for hypertension, diabetes, and myocardial infarction in his mother. The patient denied any tobacco, ethanol, or illicit drug use.

**Figure 1:**
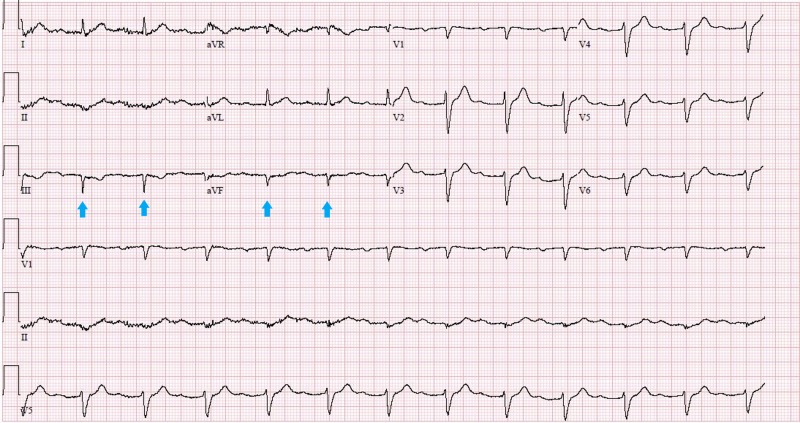
EKG on admission showing first-degree heart block, intraventricular conduction delay with a QRS of 136 milli-seconds and pathological Q waves in inferior leads (blue arrows). EKG, electrocardiogram.

Blood tests showed elevated concentrations of troponin I (0.32 ƞg/ml; normal < 0.04 ƞg/ml) and brain-type natriuretic peptide (186 pg/ml; normal < 101 pg/ml). His complete blood count and chemistry panel were otherwise unremarkable. An electrocardiogram (EKG) revealed a first-degree heart block and intra-ventricular conduction delay, with a QRS of 136 milli-seconds and pathological Q waves in the inferior leads (Figure 1). He was diagnosed with non-ST elevation myocardial infarction (NSTEMI) and was administered dual antiplatelets therapy and heparin.

**Figure 2:**
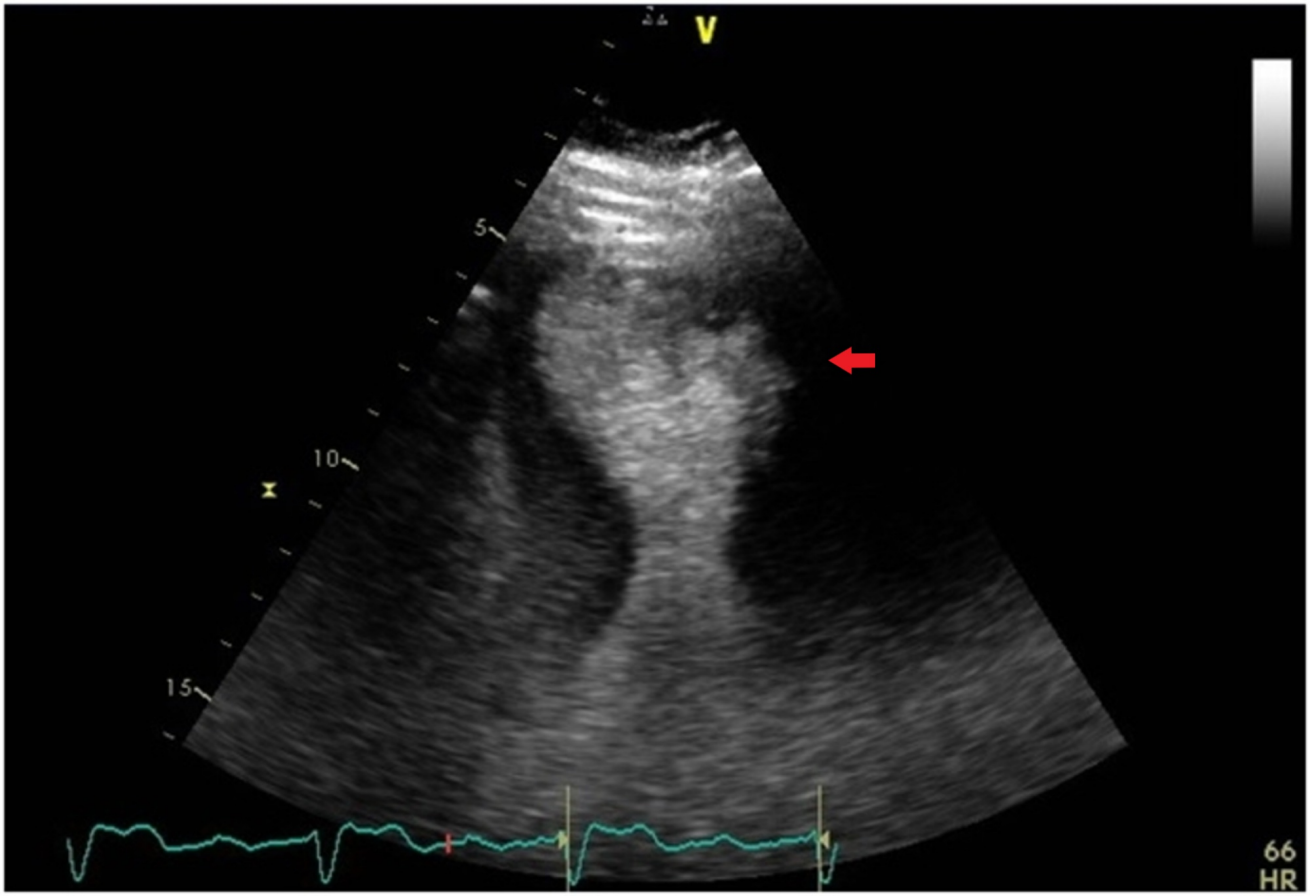
Apical four chamber view showing apical and periapical akinesia and dilation (red arrow) along with preservation of the basilar segment. Moderately increased left ventricular wall thickness was noted too.

**Figure 3:**
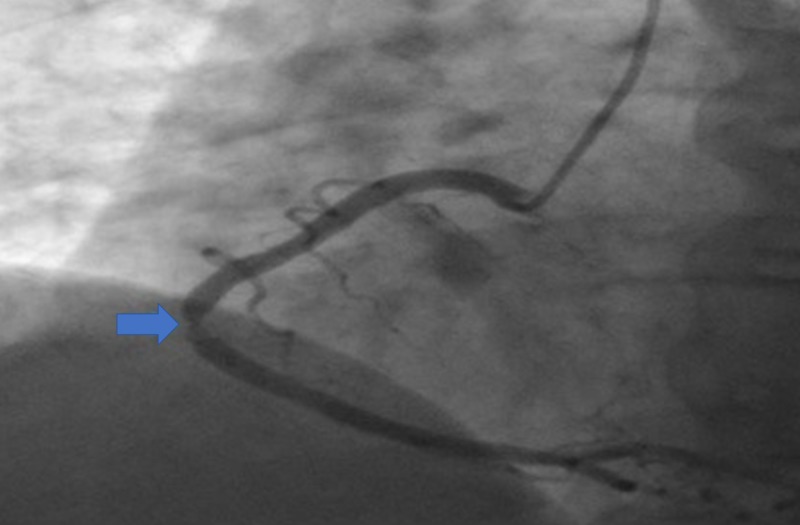
Coronary angiogram showing 90% stenosis in the mid segment of the right coronary artery (blue arrow).

Echocardiography revealed impaired left ventricular systolic function of 30%-35%. Contrast-enhanced imaging of the left ventricle showed evidence of apical and peri-apical and mid to apical anterolateral akinesia and dilation along with preservation of the basilar segment (Figure 2). These echocardiographic findings were indicative of TCM and left anterior descending (LAD) artery occlusion. Coronary angiogram revealed 90% stenosis in the middle segment of the RCA (Figure 3), with a thrombolysis in myocardial infarction (TIMI) flow of II. However, no significant stenotic lesions were found in the LAD and circumflex arteries. A drug-eluting stent was inserted into the RCA, resulting in 0% residual stenosis and a TIMI flow of III.

**Figure 4:**
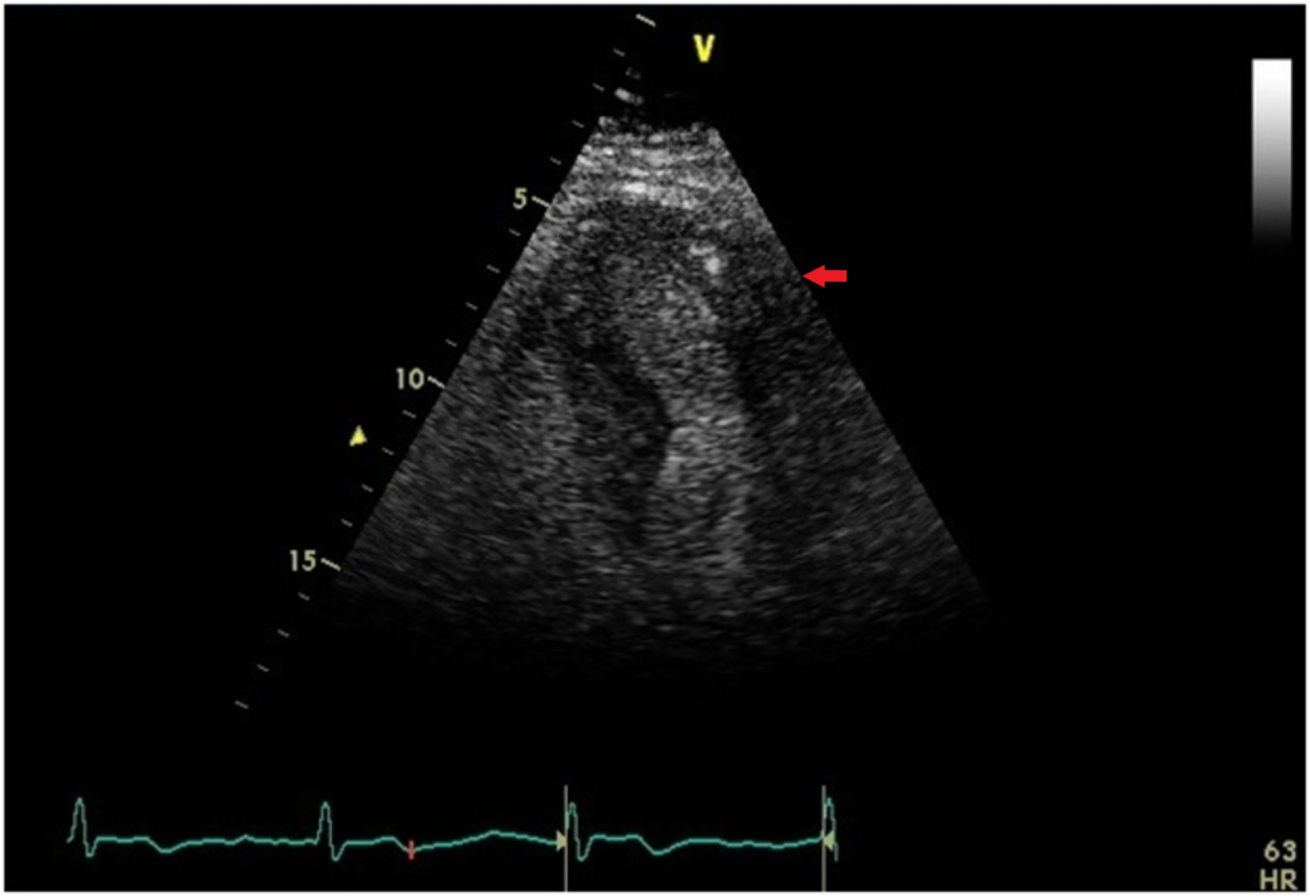
Apical four chamber view showing resolution of apical and periapical akinesia and dilation (red arrow).

The patient was discharged home on guideline-directed medical therapy. Repeat echocardiography four weeks later demonstrated resolution of the wall motion abnormalities along with restoration of normal left ventricular ejection fraction (Figure 4).

## Discussion

This report describes a patient with simultaneous NSTEMI and TCM. Although no causal relationship could be determined, the co-occurrence of NSTEMI and TCM suggests a possible association between ACS and TCM. Although our patient did not present with the classic ST depression of NSTEMI, he demonstrated other clinical characteristics of NSTEMI, including chest pain typical of cardiac origin; risk factors, including hypertension, diabetes mellitus, and hyperlipidemia; elevated troponin level; and pathological Q waves in the inferior leads, which may indicate established myocardial injury and possible infarction in the RCA. Coronary angiogram was, therefore, deemed necessary. Although angiography did not show complete occlusion of the RCA (90% stenosis), the TIMI flow was reduced to II. Along with the presence of Q waves in the inferior leads, these factors suggested possible ruptured plaque and lysis. Stenting resulted in the complete resolution of stenosis, with TIMI flow increasing from II to III following stent deployment.

The Mayo Clinic criteria defined the elements needed to diagnose TCM. These criteria include the absence of occlusive CAD or acute plaque rupture on angiography, the presence of a reversible or transient wall motion abnormality not limited to a single vascular territory, and EKG changes in the absence of pheochromocytoma, myocarditis, or intracranial bleeding. These and other criteria, including the Johns Hopkins criteria, have emphasized the absence of acute CAD that can explain the wall motion abnormalities observed in patients with TCM, such as LAD abnormalities that can explain classic apical regional wall motion abnormalities [2]. As these criteria were first proposed in 2004, the number of reports of coexistent CAD in patients with TCM has increased [4-7]. For example, a study of 97 Japanese patients with TCM reported significant CAD in 10% of these patients, with the left circumflex and LAD being the most commonly affected coronary arteries [4]. Involvement of the LAD may make it difficult to determine whether the arterial disease or TCM is responsible for the apical ballooning, especially in cases of spontaneous clot lysis. Cardiac magnetic resonance imaging may be able to differentiate between these conditions [8] in that late gadolinium enhancement (LGE) is indicative of necrosis and, hence, infarction, whereas an absence of LGE is a hallmark of TCM.

Despite evidence suggesting that incidental nonacute CAD occurs in a significant percentage of patients with TCM, it is not yet clear whether ACSs can trigger or are associated with TCM, especially in patients with wall motion abnormalities extending beyond the culprit vessel and in those with systolic dysfunction being disproportionately reduced compared with the degree of vascular insult. For example, a report describing an 88-year-old woman with an occluded diagonal branch, acute plaque rupture, and echocardiographic findings of apical akinesia extending beyond the occluded vascular territory suggested that this form of ACS may have triggered TCM through somatic stress [9]. Another report of a 70-year-old woman who presented with ST-elevation myocardial infarction due to an occluded drug-eluting stent in the RCA found that this patient had typical TCM wall motion abnormalities of the apex and midventricular region [7].

Post-ischemic myocardial stunning can also present with regional wall motion abnormalities similar to TCM, suggesting that TCM may actually be a form of stunning [10]. Because stunning is generally limited to the affected ischemic area, this cannot explain how TCM can involve remote myocardial areas, as in our patient, in whom the RCA was stenotic, and the TCM involved the LAD territory.

The behavior of the remote noninfarcted myocardium in patients with myocardial infarction is a complex process. The remote myocardium may become hyperkinetic and contractile during and after acute myocardial infarction, possibly due to increased catecholamine levels [11]. In contrast, the involvement of the RCA, as in our patient, was accompanied by reduced function and akinesia of the remote myocardium at the left ventricular apex and mid to apical anterolateral wall. These findings cast doubt on the two possible opposite effects of increased local levels of catecholamines, causing hyperkinesia in one case and hypokinesia in the other.

## Conclusions

In conclusion, findings in our patient and in similar patients showed no causal relationship between ACS and TCM. However, ACS, like any other physical illness, may trigger TCM. Further aspects of the pathogenesis of this reversible condition and the possible association between ACS and TCM have not yet been determined.

## References

[1] Vitale C, Rosano GM, Kaski JC (2016). Role of coronary microvascular dysfunction in Takotsubo cardiomyopathy. Circ J.

[2] Madias JE (2014). Why the current diagnostic criteria of Takotsubo syndrome are outmoded: a proposal for new criteria. Int J Cardiol.

[3] Pelliccia F, Kaski JC, Crea F, Camici PG (2017). Pathophysiology of Takotsubo syndrome. Circulation.

[4] Kurisu S, Inoue I, Kawagoe T (2009). Prevalence of incidental coronary artery disease in Tako-tsubo cardiomyopathy. Coron Artery Dis.

[5] Koeth O, Zeymer U, Schiele R, Zahn R (2010). Inferior ST-elevation myocardial infarction associated with Takotsubo cardiomyopathy. Case Rep Med.

[6] Gaibazzi N, Ugo F, Vignali L, Zoni A, Reverberi C, Gherli T (2009). Tako-Tsubo cardiomyopathy with coronary artery stenosis: a case-series challenging the original definition. Int J Cardiol.

[7] Tota F, Ruggiero M, Sassara M (2013). Subacute stent thrombosis and stress-induced cardiomyopathy: trigger or consequence?. Am J Cardiovasc Dis.

[8] Syed IS, Prasad A, Oh JK (2008). Apical ballooning syndrome or aborted acute myocardial infarction? Insights from cardiovascular magnetic resonance imaging. Int J Cardiovasc Imaging.

[9] Redfors B, Råmunddal T, Shao Y, Omerovic E (2014). Takotsubo triggered by acute myocardial infarction: a common but overlooked syndrome?. J Geriatr Cardiol.

[10] Y-Hassan S (2015). Post-ischemic myocardial stunning was the starting point of Takotsubo syndrome: restitution is justified after falling down on. Int J Cardiol.

[11] Rechavia E, de Silva R, Nihoyannopoulos P, Lammertsma AA, Jones T, Maseri A (1995). Hyperdynamic performance of remote myocardium in acute infarction. Correlation between regional contractile function and myocardial perfusion. Eur Heart J.

